# Delineating Host–Guest–Solvent Interactions in Solution from Gas-Phase Host–Guest Configurations: Thermodynamic Reversal and Structural Correlation of 24-Crown-8/H^+^/Diaminopropanol Non-Covalent Complexes in Aqueous Solution vs. in the Gas Phase

**DOI:** 10.3390/molecules30081723

**Published:** 2025-04-11

**Authors:** Young-Ho Oh, So Yeon Lee, Han Bin Oh, Sungyul Lee

**Affiliations:** 1 Department of Applied Chemistry, Kyung Hee University, 1732, Deogyeong-daero, Giheung-gu, Yongin-si 17104, Republic of Korea; 2 Department of Chemistry, Sogang University, 35 Baekbeom-ro, Mapo-gu, Seoul 04107, Republic of Korea

**Keywords:** host–guest, crown ether, diaminopropanol, structural correlation

## Abstract

We study the structures of 24-crown-8/H^+^/diaminopropanol (CR/DAPH^+^) and 24-crown-8/CsF/H^+^/diaminopropanol (CR/CsF/DAPH^+^) non-covalent host–guest complexes in both the gas phase and aqueous solution using the density functional theory (DFT) method. We examine the environment (complexation with CR vs. solvation) around the guest functional groups (ammoium, hydroxyl, and amino) in the CR/DAPH^+^ and CR/CsF/DAPH^+^ complexes. We find that the gas-phase configurations with the ‘naked’ hydroxyl/amino devoid of H-bonding with CR or CR/CsF are structurally correlated with the lowest Gibbs free energy conformers in aqueous solution in which the functional groups are solvated off the CR or CR/CsF host. We predict that the latter thermodynamically disadvantageous host–guest configurations would be identified in the gas phase by infrared multiphoton dissociation (IRMPD) spectroscopy, originating from the complexes in aqueous solution. This predicted ‘thermodynamic reversal’ and ‘structural correlation’ of the host–guest configurations in the gas phase vs. in solution are discussed in relation to the possibility of obtaining information on host–guest–solvent interactions in the solution phase from the gas-phase host–guest configurations.

## 1. Introduction

As a technique for the selective production of non-covalent ionic host–guest complexes [1,2,3,4,5,6,7,8,9] in a gas phase, electrospray ionization/mass spectroscopy [10,11,12,13] (ESI/MS) has proven to be highly pivotal. The differentiation of a guest molecule by a chiral host [14,15,16,17,18] has been one of the most intensively studied topics in host–guest chemistry, aimed at shedding light on the processes of molecular recognition and self-assembly. Determining the structures of host–guest pairs in the gas phase is often challenging, but infrared multiple photon dissociation (IRMPD) spectroscopy [14,19,20,21,22,23,24,25,26,27,28] has been shown to be a powerful tool to elucidate the features of non-covalent interactions in gas-phase complexes. When combined with quantum chemical calculations, IRMPD spectra of biomolecules bound to a macrocycle host can reveal the detailed interactions in the host–guest pairs, as exemplified in our previous studies [29,30,31,32] of protonated AAH^+^ (AA = alanine, isoleucine, lysine) bound to the permethylated *β*-cyclodextrin (perm-CD) host in the gas phase.

**Scheme 1:**
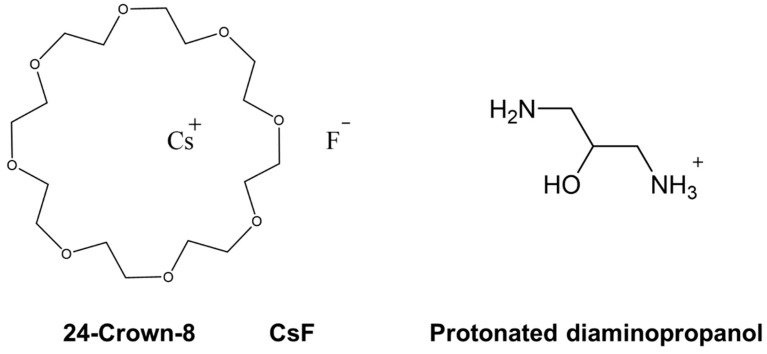
Components of 24-Crown-8/protonated diaminopropanol and 24-Crown-8/CsF/protonated diaminopropanol covalent host–guest complexes.

Here, we theoretically examine the 24-crown-8/H^+^/diaminopropanol (CR/DAPH^+^) and 24-crown-8/CsF/H^+^/diaminopropanol (CR/CsF/DAPH^+^) complexes (Scheme 1) both in the gas phase and in aqueous solution to examine the structural connectivity of the non-covalent host–guest complexes in the two phases. We probe the environment (complexation with CR vs. solvation) around the guest functional groups (ammonium, hydroxyl, and amino) in the CR/DAPH^+^ complex, illustrating that the ammonium only binds at the CR host, whereas both -OH and -NH_2_ are solvated by water molecules off the host. We also study the CR/CsF/DAPH^+^ host–guest system to propose a new scheme of introducing F^−^ as a ‘probe’ for determining (by ^19^F-NMR spectroscopy) the environment (specifically, complexation vs. solvation) around the functional groups in the guest in solution, which is very difficult to observe by ^1^H-NMR spectroscopy due to the very rapid proton/proton and proton/deuterium exchange and the consequent extreme broadening of the proton peaks. Since the H-bonds between F^−^ and the guest hydroxyl/ammonium groups would be stronger than those with the CR host, using F^−^ is also anticipated to give a more salient case of thermodynamic reversal and a more conspicuous example of the structural correlation [31] of host–guest pairs in solution and in the gas phase. We find that the hydroxyl (and/or ammonium) in the DAPH^+^ guest chooses to be solvated by water molecules in the solution phase, whereas in the lowest Gibbs free energy gas-phase conformers, it prefers complexation with CR/CsF. We compare these findings with those for the CR/DAPH^+^ complex (which is devoid of CsF) to show that the host–guest interactions in the CR/CsF/DAPH^+^ system are very similar to those in the CR/DAPH^+^ complex, so that the influence of (perturbations due to) the presence of the CsF probe is minimal, in that the CR/CsF host behaves very much like the CR unit.

We also predict that the thermodynamic stability of the (CR/DAPH^+^) and (CR/CsF/DAPH^+^) host–guest configurations in the gas phase is reversed in aqueous solution (‘thermodynamic reversal’) [32], and that the lowest Gibbs free energy conformer with solvated (without interactions with the CR or CR/CsF host) guest functional groups in solution is structurally related [32] to the thermodynamically disadvantageous gas-phase complex with the ‘naked’ -OH (and/or -NH_2_). From these observations, we consolidate our previous proposal [32] that the gas-phase conformers of the CR/DAPH^+^ and CR/CsF/DAPH^+^ non-covalent complexes produced and to be identified by the ESI/MS and IRMPD procedure, respectively, may originate from the thermodynamically most favored host–guest complexes in solution, and that this close structural connection implicates the possibility of using the ESI/MS/IRMPD protocol for elucidating host–guest–solvent interactions in the solution phase directly from host–guest configurations in the gas phase.

## 2. Results

### 2.1. Structures and Thermodynamic Stability of CR/DAPH^+^ Non-Covalent Complex in Aqueous Solution

**Figure 1:**
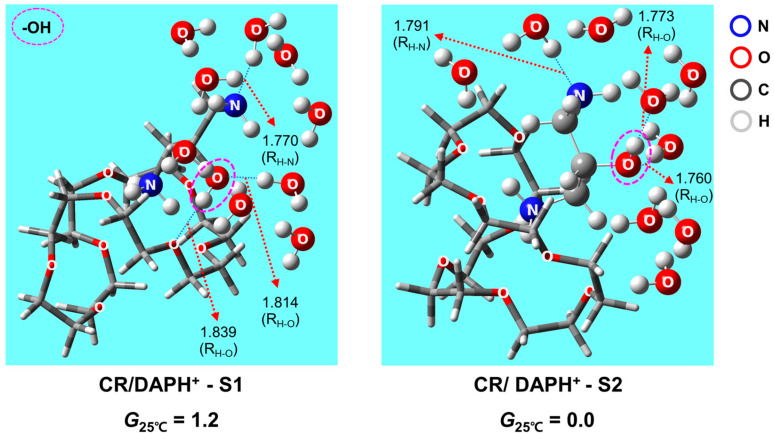
Calculated structures of 24-Crown-8/DAPH^+^ in aqueous solution. Relative Gibbs free energy in kcal/mol and distance in Å. Blue background represents solvent continuum.

Figure 1 (For Cartesian coordinates, see Supplementary) depicts the calculated lower Gibbs free energy structures (CR/DAPH^+^-S1, CR/DAPH^+^-S2) of the CR/DAPHCl complex in aqueous solution. We find that nine water molecules completely envelop the DAPH^+^ guest in the first shell; thus, we employ the (CR/DAPH^+^ + 9 H_2_O + water continuum) as the model system for the complex in aqueous solution. The two conformers exhibit distinct structural features: In CR/DAPH^+^-S1, the CR host and the DAPH^+^ guest form the complex through the [CR…ammonium]/[CR…hydroxyl] H-bonds, with the amino positioned off the CR unit. In CR/DAPH^+^-S2, on the other hand, -NH_3_^+^ only interacts with the CR unit, while the hydroxyl and the amino groups are solvated away from the CR host. Since the H-bonds between the two guest functional groups (-OH and -NH_2_) and the water molecules are stronger than those with the CR host, the lower (by 1.2 kcal/mol) Gibbs free energy of the CR/DAPH^+^-S2 conformer is easily understood.

### 2.2. Structures and Thermodynamic Stability of CR/CsF/DAPHCl in Aqueous Solution

The use of the 24-crown-8/CsF/DAPH^+^ host–guest system is motivated by two presumptions: First, it may allow the examination of the environment (solvation vs. complexation) around the DAPH^+^ guest functional groups by ^19^F-NMR spectroscopy (observing the characteristic positions (chemical shifts) of ^19^F-NMR peaks), as demonstrated in our previous investigation [33]. Second, the [F^−^…NH_3_^+^-], [F^−^…HO-], and/or [-OH…F^−^…NH_3_^+^-] H-bonds, which are stronger than the corresponding [CR…NH_3_^+^-], [CR…HO-], and/or [-OH…CR…NH_3_^+^-] H-bonds, respectively, are expected to ‘magnify’ the reversal of the thermodynamic stability of the solution-phase host–guest complex vs. that in the gas phase, as discussed below. Thus, by presenting theoretical analysis of the CR/CsF/DAPH^+^ system, we propose a scheme (^19^F-NMR spectroscopy for probing the [F^−^…NH_3_^+^-], [F^−^…HO-], [-OH…F^−^…NH_3_^+^-], and [F^−^…solvent] hydrogen bonds) for the unambiguous experimental vindication of the structural connectivity of host–guest configurations in solution to those in the gas phase.

**Figure 2:**
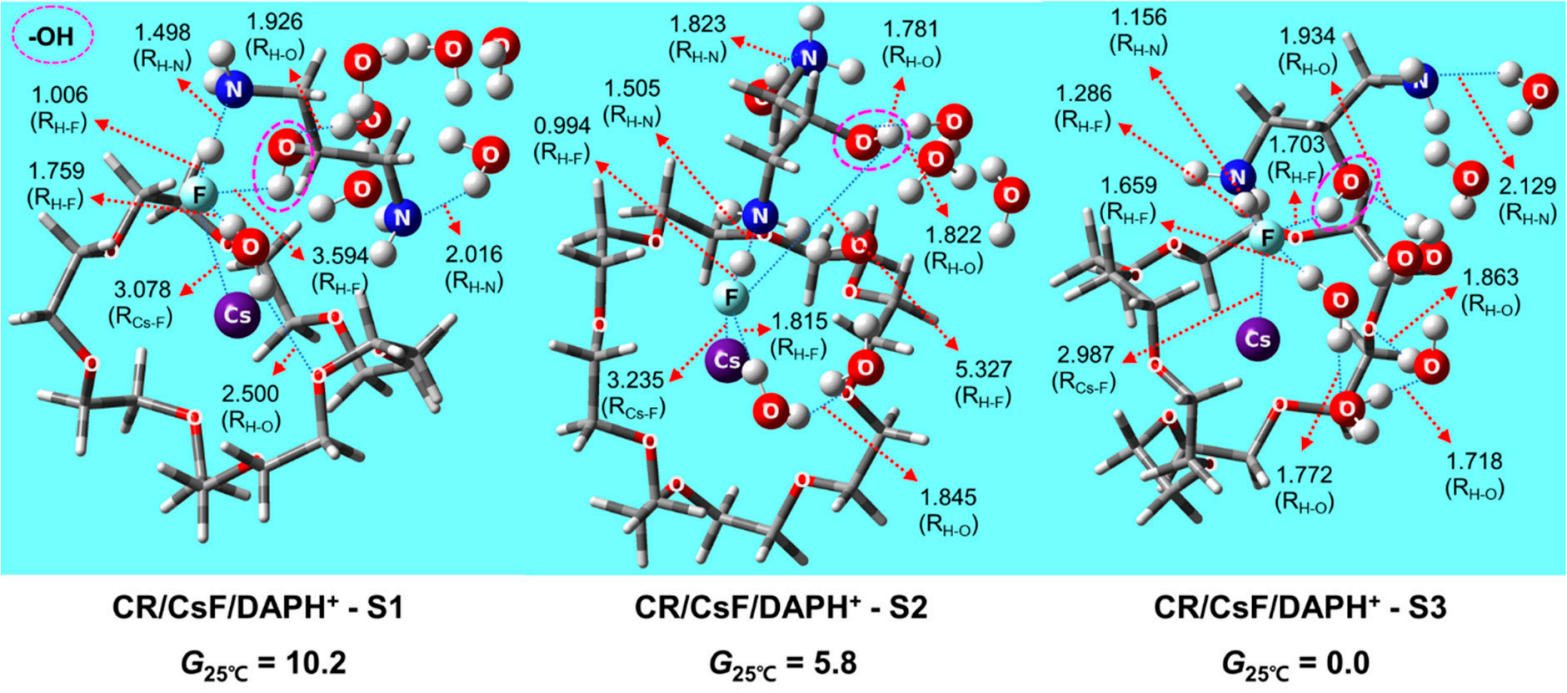
Calculated structures of 24-Crown-8/CsF/DAPHCl in aqueous solution. Relative Gibbs free energy in kcal/mol and distance in Å. Blue background represents solvent continuum.

Figure 2 (For Cartesian coordinates, see Supplementary) illustrates the calculated structures (CR/CsF/DAPH^+^-S1, CR/CsF/DAPH^+^-S2, and CR/CsF/DAPH^+^-S3) of the 24-Crown-8/CsF/DAPHCl complex in solution, modeled as the supramolecule CR/CsF/DAPHCl/(H_2_O)_7_ in water continuum for the reason discussed below. In all structures, NH_3_^+^ is the main contact on the CR/CsF host. The conformers, however, differ in the interactions between the two other functional groups (-OH and -NH_2_) and the CR/CsF unit. In CR/CsF/DAPH^+^-S1, NH_3_^+^ forms a H-bond with F^−^, whereas the two other guest functional groups (-OH and -NH_2_) interact with the O atoms in the CR ring. In the CR/CsF/DAPH^+^-S2 conformer, NH_3_^+^ forms H-bonds both with F^−^ and the CR unit, while -OH and -NH_2_ are solvated off the CR/CsF host. In CR/CsF/DAPH^+^-S3, NH_3_^+^ interacts with F^−^ and with the CR ring, the hydroxyl interacts with the solvent and with F^−^, and the amino is fully solvated off the CR/CsF host. These strong H-bonds between the guest functional groups and the CR/solvent seem to be the origin of high thermodynamic advantage (Gibbs free energy lower than CR/CsF/DAPH^+^-S1 and CR/CsF/DAPH^+^-S2 by 10.2 and 5.8 kcal/mol, respectively). Compared with the CR/DAPH^+^ conformers presented in Figure 1, it can be seen that the structures of the lowest Gibbs free energy configurations of the CR/CsF/DAPH^+^ and CR/DAPH^+^ systems are very similar in that the two guest functional groups (hydroxyl and amino) are solvated by water molecules. On the other hand, the differences in the Gibbs free energies of the most favored host–guest configurations relative to other conformers are calculated to be ‘magnified’. These two observations may validate the use of the CsF ‘probe’ for determining the features of H-bonding between the CR host and the DAPH^+^ guest in solution, which may be achievable by ^19^F-NMR spectroscopy [33].

### 2.3. Structures and Relative Gibbs Free Energy of CR/DAPH^+^ in the Gas Phase

**Figure 3:**
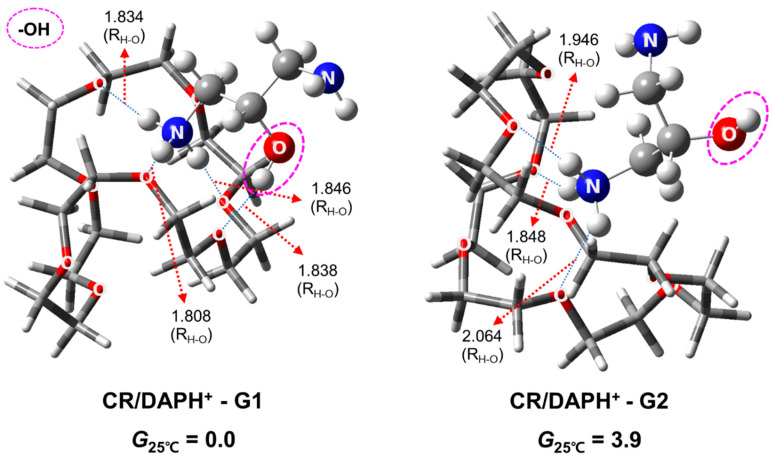
Calculated lowest Gibbs free energy structures of 24-Crown-8/DAPH^+^ in the gas phase. Relative Gibbs free energy (at 25 °C) in kcal/mol and distance in Å.

Figure 3 (For Cartesian coordinates, see Supplementary) depicts the calculated lowest Gibbs free energy structures of the CR/DAPH^+^ complex in the gas phase obtained by extensive searching over the landscape of the potential surface. CR/DAPH^+^-G1 and CR/DAPH^+^-G2 structurally correspond to the host–guest configuration CR/DAPH^+^-S1 and CR/DAPH^+^-S2 in solution, respectively, depicted in Figure 1. It is highly intriguing to observe that the gas-phase Gibbs free energy of CR/DAPH^+^-G2, which is structurally connected to the lowest Gibbs free energy solution-phase complex CR/DAPH^+^-S2, is now the highest (higher than that of CR/DAPH^+^-G1 by 3.9 kcal/mol), illustrating a ‘thermodynamic reversal’ [32] of 5.1 kcal/mol. In the most stable solution-phase complex CR/DAPH^+^-S2, the hydroxyl, which was fully surrounded by nine explicit H_2_O molecules in the first shell around the complex, becomes ‘naked’ in the gas phase. This ‘thermodynamic reversal’ was the basis of the proposed possibility of elucidating the host–guest–solvent interactions from the host–guest configurations in the gas phase that is connected with the solution phase by the ESI/MS procedure [32].

**Figure 4:**
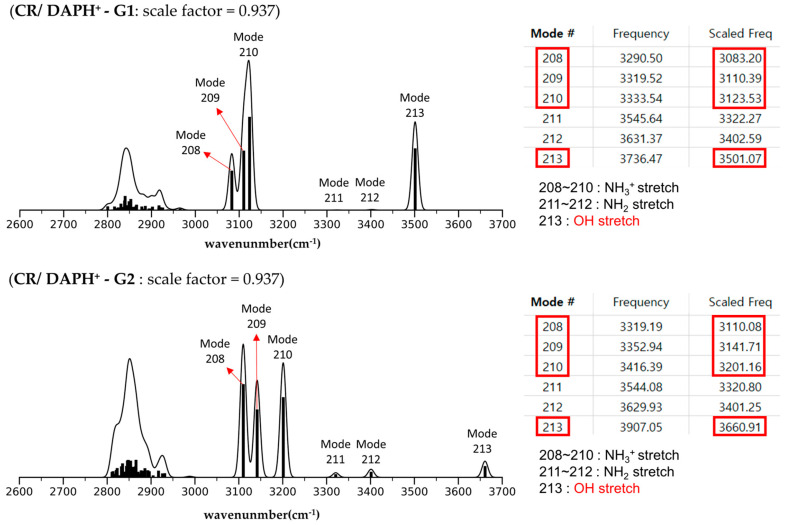
Calculated IR spectra of gas-phase 24-Crown-8/DAPH^+^ in the gas phase. Frequency in cm^−1^.

These structural features are also manifested in the corresponding infrared spectra (Figure 4): The strong band at 3661 cm^−1^ indicates the absorption of the isolated -OH in CR/DAPH^+^-G2, whereas the band for CR/DAPH^+^-G1 at 3501 cm^−1^ corresponds to the red-shifted -OH, indicating the formation of H-bonds between -OH and the CR unit. The strong red-shifts (3080~3200 cm^−1^) of the -NH stretch modes of the ammonium in the two complexes illustrate the strong interactions between -NH_3_^+^ and the CR host. The -NH stretch modes of the amino in the 3320~3400 cm^−1^ region signify the isolated -NH_2_ in the two configurations. These IR spectra are to be compared with the experimental IRMPD spectra for structural identification of the gas-phase CR/CsF/DAPH^+^ complex generated from the solution phase by the ESI/MS procedure.

### 2.4. Structures and Relative Gibbs Free Energy of CR/CsF/DAPH^+^ in the Gas Phase

**Figure 5:**
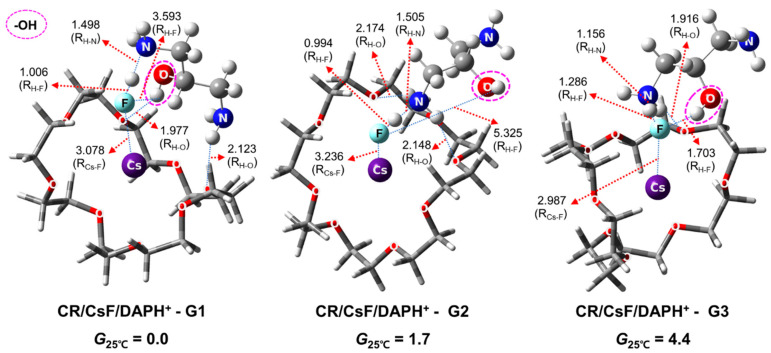
Calculated lower Gibbs free energy structures of the gas-phase 24-Crown-8/CsF/DAPH^+^. Relative Gibbs free energy (at 25 °C) in kcal/mol and distance in Å.

Figure 5 (For Cartesian coordinates, see Supplementary) presents the calculated gas-phase structures of the CR/CsF/DAPH^+^ complex (modeled as CR/DAPH^+^ + 7 H_2_O + water continuum) with distinct structural features. In the lowest Gibbs free energy (at 25 °C) configuration CR/CsF/DAPH^+^-G1, the [F^−^…-NH_3_^+^], [-OH…CR], and [NH_2_…CR] J H-bonds (*R*_F-HN_ = 1.006 Å, *R*_OH-O_ = 1.977 Å, and *R*_NH-O_ = 2.123 Å, respectively) provide the thermodynamic advantage, while the hydroxyl does not interact with F^−^. On the other hand, in the thermodynamically less favorable CR/CsF/DAPH^+^-G2, NH_3_^+^ forms H-bonds both with F^−^ and CR (*R*_F-HN_ = 0.994 Å; *R*_NH-O_ = 2.174 Å), while -OH and -NH_2_ are positioned off the CR/CsF host without interactions. In the highest relative Gibbs free energy structure, the ammonium and hydroxyl form H-bonds with F^−^ (*R*_F-HN_ = 1.286 Å; *R*_OH-O_ = 1.703 Å), whereas the amino is completely isolated.

For the present CR/CsF/DAPH^+^ system, we also predict a ‘thermodynamic reversal’ in the relative CR/CsF/DAPH^+^-G1 Gibbs free energies of the thermodynamically most favorable complex in the gas phase (CR/CsF/DAPH^+^-G1) versus that in aqueous solution (CR/CsF/DAPH^+^-S1) by as much as 10.2 kcal/mol. Thus, the presence of CsF indeed seems to result in a more salient ‘thermodynamic reversal’ than that observed for the CR/DAPH^+^ system.

**Figure 6:**
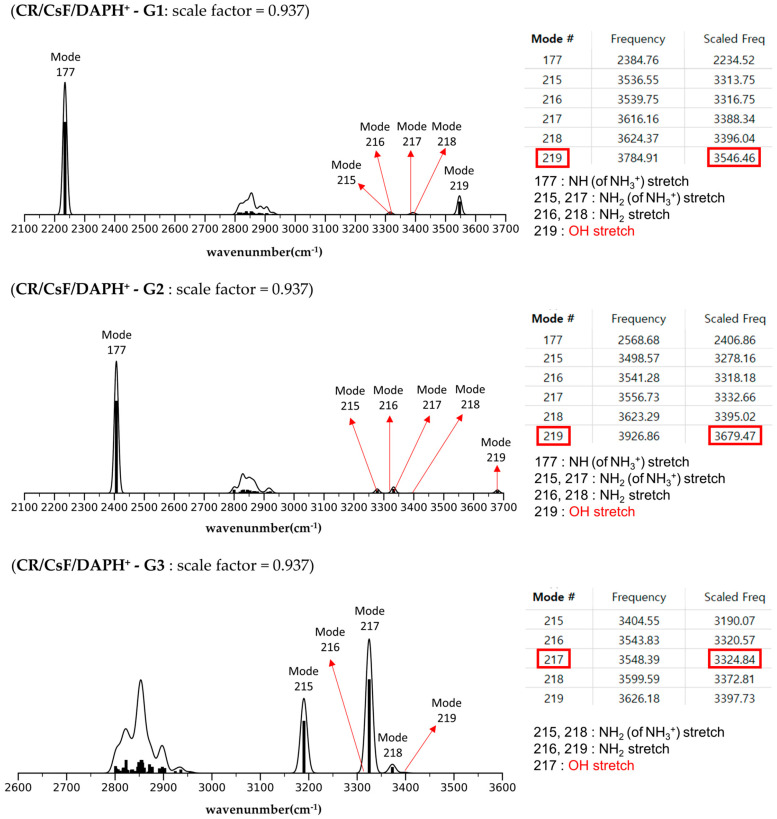
Calculated IR spectra of 24-Crown-8/CsF/DAPH^+^ in the gas phase. Frequency in cm^−1^.

Figure 6 presents the calculated IR spectra of the CR/CsF/DAPH^+^ complex in the gas phase, which can be interpreted by these structural features of the gas-phase configurations. The -OH stretch frequencies are the most conspicuous feature, as they may represent the environment of the guest functional group, specifically concerning the presence/absence of the H-bonding with F^−^ and/or with the CR ring. The red-shifted frequencies of 3546, and 3325 cm^−1^ for CR/CsF/DAPH^+^-G1 and CR/CsF/DAPH^+^-G3, respectively, are due to the [-OH…CR] and [-OH… F^−^] H-bonds, whereas the 3679 cm ^−1^ band for the CR/CsF/DAPH^+^-G2 conformer represents the ‘naked’ OH.

The low-frequency ammonium N-H stretch modes (#177 (2235 cm^−1^) and #177 (2407 cm^−1^) for CR/CsF/DAPH^+^-G1 and CR/CsF/DAPH^+^-G2, respectively) deserve scrutiny. In these two configurations, the ammonium H is almost transferred to F^−^, so that the elongated N-H bonds (1.498 and 1.505 Å) give rise to very low IR frequencies. In CR/CsF/DAPH^+^-G3, on the other hand, the N-H bond is short (1.156 Å), and the stretch of this strong bond appears at a higher frequency (>3200 cm^−1^). The -NH_2_ stretch bands for the three CR/CsF/DAPH^+^ configurations are of low intensity and appear at similar positions (~3313 and ~3396 cm^−1^), indicating that they are either isolated or in very weak interaction with the CR ring (but not with F^−^). Comparison of these IR spectra with the experimental IRMPD spectra would lead to an unambiguous identification of the CR/CsF/DAPH^+^ complex in the gas phase.

**Figure 7:**
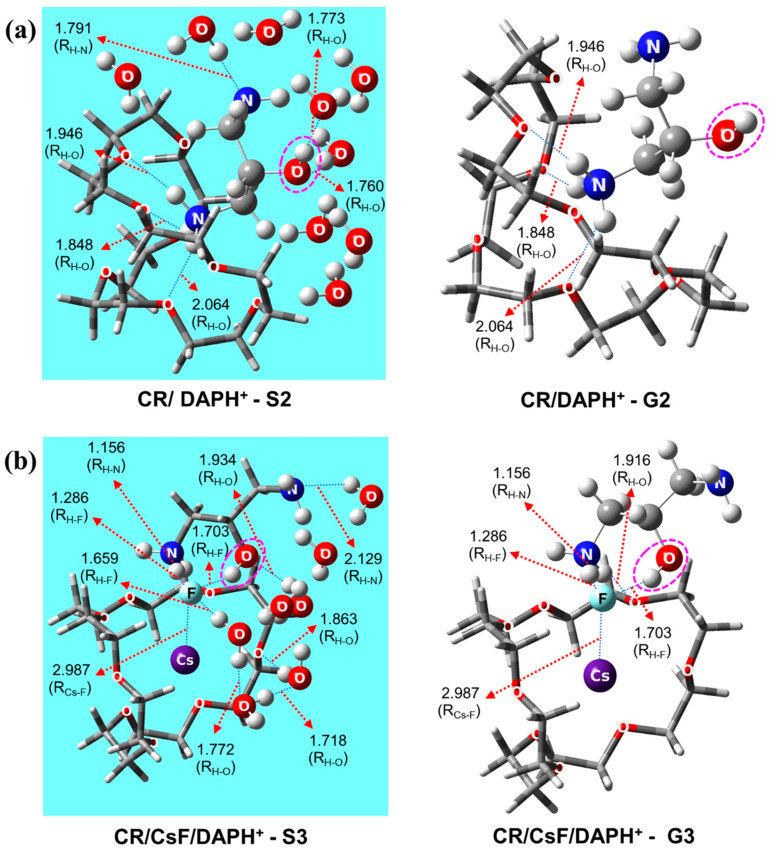
Comparison of the lowest Gibbs free energy for (**a**) 24-Crown-8/DAPH^+^ and (**b**) 24-Crown 8/CsF/DAPH^+^ configurations in solution with their structurally correlating gas-phase structures. Blue background represents the solvent continuum.

Finally, Figure 7 depicts the structural connectivity of CR/DAPH^+^-S2 and CR/DAPH^+^-G2, and of CR/CsF/DAPH^+^-S3 and CR/CsF/DAPH^+^-G3. The host–guest configurations in solution and in the gas phase are shown to be very similar.

Based on the present findings, the most relevant question concerning the gas-phase CR/DAPH^+^ and CR/CsF/DAPH^+^ complexes would be the following: which conformers of the gas-phase CR/DAPH^+^ and CR/CsF/DAPH^+^ complexes are to be observed and identified by the IRMPD technique? In our previous studies, it was demonstrated that the relative thermodynamic stability (relative Gibbs free energy) of the gas-phase host–guest complexes may not determine the populations of the complexes if the gas-phase environment produced in the ESI/MS experiments is not in thermal equilibrium. In this situation, the most stable host–guest configurations in the solution phase may give rise to the structures of the host–guest in the gas phase.

If the lowest Gibbs free energy CR/DAPH^+^ and CR/CsF/DAPH^+^ complexes in solution are stripped off the solvent molecules by the ESI/MS, and if the produced gas-phase host–guest complexes are detected in the gas phase before thermal equilibrium is attained, then the thermodynamically least favorable (highest Gibbs free energy) CR/DAPH^+^-G2 and CR/CsF/DAPH^+^-G3 with the “naked” -OH and -NH_2_ would be observed in the gas phase as produced by the ESI/MS protocol, originating from the thermodynamically most favorable solution-phase configurations CR/DAPH^+^-S2 and CR/CsF/DAPH^+^-S3.

We may now discuss the implications of the present findings with regard to the ESI/MS/IRMPD experiments for host–guest complexes. If the proposition from our previous works on the perm-CD/L-AAH^+^ (AA = Ala, Ile, Lys) system [29,30,31,32,34,35,36] also applies to our present 24-Crown-8/DAPH^+^ and 24-Crown-8/CsF/DAPH^+^ host–guest complexes, then thermodynamically, the most stable solution-phase complex CR/DAPH^+^-S2 [CR/CsF/DAPH^+^-S3] would be instantly ‘frozen’ into CR/DAPH^+^-G2 [CR/CsF/DAPH^+^-G3] in the gas phase, produced under thermal non-equilibrium conditions by the ESI/MS techniques. Thus, the identification of the gas-phase host–guest complexes CR/DAPH^+^-G2 and CR/CsF/DAPH^+^-G3, which are thermodynamically least favorable, would further consolidate our proposition of the structural correlation between the host–guest complexes in solution and in the gas phase, and of the possibility of obtaining information on host–guest–solvent interactions in solution from host–guest structures in the gas phase via the ESI/MS/IRMPD protocol.

## 3. Computational Details

All calculations were performed using the wB97X-D functional [34], which may treat weak interactions (including hydrogen bonding) effectively, with the 6-311G** basis set implemented in the Gaussian16 suite of programs [35]. For the complexes in aqueous solution, the supramolecule/continuum approach was adopted, treating the solvent molecules directly interacting with the guest functional groups in the first shell around the complex as explicit molecules (CR/DAPH^+^/(H_2_O)_n_, CR/CsF/DAPH^+^(H_2_O)_n_), and other numerous H_2_O molecules in the second shell and beyond as a water continuum modeled by the SMD method [36]. A scale factor of 0.927 was used to fit the calculated IR frequency of the hydroxyl and carboxyl -OH stretch modes to the experimental value (3660 cm^−1^) [37]. All structures were obtained by verifying that all vibrational frequencies were real.

## 4. Conclusions

We have presented the calculated structures of the 24-Crown-8/DAPH^+^ and 24-Crown-8/CsF/DAPH^+^ complexes in both the gas phase and solution. Our main focus was on exploring the structural connectivity of the complexes in the two phases. We have proposed that the most stable (lowest Gibbs free energy) configurations of the complexes in the solution phase might produce higher Gibbs free energy host–guest complexes in the gas phase under thermal non-equilibrium gas-phase environments via the ESI/MS procedure, whose structures could subsequently be identified by the IRMPD technique. We also provided a theoretical basis for using F^−^ as a ‘probe’ for determining the conformation of the guest functional groups. We believe that these findings offer further support for our approach of characterizing host–guest–solvent interactions in solution by using the gas-phase structures of host–guest complexes. We are working on unambiguous experimental validation for this proposition of structural correlation between the host–guest configurations in solution and in the gas phase. Further studies of these fascinating macrocycle/biomolecule host–guest systems would be an extremely intriguing avenue for exploration.

## Data Availability

The original contributions presented in this study are included in the article. Further inquiries can be directed to the corresponding authors.

## Supplementary Materials

The following supporting information can be downloaded at: https://www.mdpi.com/article/10.3390/molecules30081723/s1.
