# Supplementary material for: Delineating Host–Guest–Solvent Interactions in Solution from Gas-Phase Host–Guest Configurations: Thermodynamic Reversal and Structural Correlation of 24-Crown-8/H+/Diaminopropanol Non-Covalent Complexes in Aqueous Solution vs. in the Gas Phase
Source: Molecules. 2025 Apr 11;30(8):1723. doi: 10.3390/molecules30081723 (PMC12029154; doi:10.3390/molecules30081723)
Supplement: Supplementary file 1 [file molecules-30-01723-s001.zip › molecules-3574986-supplementary.pdf]

Delineating Host-Guest-Solvent Interactions in Solution from Gas Phase Host-Guest Configurations: Thermodynamic Reversal and Structural Correlation of 24-Crown-8/H^+/Diaminopropanol Non-Covalent Complexes in Aqueous Solution vs. in the Gas Phase

Young-Ho Oh, So Yeon Lee, Han Bin Oh and Sungyul Lee

Cartesian coordinates

CR/DAPH$^+$ - S1

**N**	**0.5787570000**	**0.7211290000**	**-0.8046660000**
**C**	**-3.2281170000**	**0.3663000000**	**-1.0205000000**
**C**	**-1.7907480000**	**0.0017560000**	**-0.6469850000**
**C**	**-0.8014010000**	**0.9690630000**	**-1.2853030000**
**H**	**-0.8026500000**	**0.8675890000**	**-2.3724140000**
**H**	**1.2436530000**	**1.3595570000**	**-1.2658800000**
**H**	**0.8498380000**	**-0.2626510000**	**-0.9614610000**
**H**	**0.6615310000**	**0.9118880000**	**0.2060220000**
**O**	**4.3035980000**	**-1.2181300000**	**1.1191070000**
**O**	**3.5580050000**	**1.1007340000**	**-0.5693750000**
**O**	**3.7133790000**	**-3.2319310000**	**-0.8524380000**
**O**	**2.1410870000**	**2.4972660000**	**-2.3899740000**
**O**	**0.9336880000**	**-2.0321890000**	**-1.3218900000**
**O**	**0.6538550000**	**3.4861020000**	**-0.1723540000**
**O**	**0.6437470000**	**-1.2963780000**	**1.4487170000**
**O**	**1.1443850000**	**1.4911500000**	**1.8907370000**
**C**	**4.2827970000**	**1.1045240000**	**0.6472240000**
**C**	**5.1467570000**	**-0.1236240000**	**0.8421380000**
**C**	**3.2922960000**	**1.7963040000**	**-2.8184890000**
**C**	**4.9408940000**	**-2.4823760000**	**1.1054220000**
**C**	**4.2851220000**	**1.5385770000**	**-1.6929700000**

**C**	**1.3114580000**	**3.7280870000**	**1.0528330000**
**C**	**0.7230910000**	**2.8286320000**	**2.1124810000**
**C**	**5.0022590000**	**-3.0915920000**	**-0.2875330000**
**C**	**0.7140300000**	**0.5999870000**	**2.9087120000**
**C**	**1.2502550000**	**-0.7805440000**	**2.6236470000**
**C**	**3.3572000000**	**-2.1977700000**	**-1.7455360000**
**C**	**0.5992180000**	**-2.9449220000**	**-0.2820210000**
**C**	**1.0743060000**	**4.2890430000**	**-1.2563880000**
**C**	**2.3658290000**	**3.8021880000**	**-1.8842360000**
**C**	**1.1904610000**	**-2.5442760000**	**1.0482380000**
**C**	**1.9449640000**	**-2.4233990000**	**-2.2404300000**
**H**	**4.9115750000**	**2.0041540000**	**0.7039500000**
**H**	**3.5317580000**	**1.1535540000**	**1.4372900000**
**H**	**5.7566720000**	**-0.3074110000**	**-0.0539690000**
**H**	**5.8358450000**	**0.0479990000**	**1.6812850000**
**H**	**3.7937080000**	**2.3270760000**	**-3.6373080000**
**H**	**2.9164700000**	**0.8486140000**	**-3.2109980000**
**H**	**5.0131160000**	**0.7899120000**	**-2.0354670000**
**H**	**4.8491260000**	**2.4481380000**	**-1.4443060000**
**H**	**1.1030680000**	**0.9315810000**	**3.8801170000**
**H**	**-0.3815140000**	**0.5925420000**	**2.9584320000**
**H**	**2.3370180000**	**-0.7508540000**	**2.4847050000**
**H**	**1.0212950000**	**-1.4294090000**	**3.4796910000**
**H**	**1.1689190000**	**4.7738300000**	**1.3578010000**
**H**	**2.3891760000**	**3.5332750000**	**0.9731820000**
**H**	**1.1917770000**	**5.3353720000**	**-0.9457510000**
**H**	**0.2723040000**	**4.2401050000**	**-1.9956320000**
**H**	**1.8122440000**	**-3.4736160000**	**-2.5225190000**
**H**	**1.7789070000**	**-1.8119270000**	**-3.1312040000**
**H**	**-0.3720540000**	**2.8863620000**	**2.0871100000**

**H**	**1.0704630000**	**3.1645690000**	**3.0979320000**
**H**	**0.9481590000**	**-3.9499800000**	**-0.5351710000**
**H**	**-0.4930090000**	**-2.9641010000**	**-0.2155570000**
**H**	**2.2730800000**	**-2.4531630000**	**0.9542350000**
**H**	**0.9530950000**	**-3.3158860000**	**1.7936900000**
**H**	**5.6465790000**	**-2.4995250000**	**-0.9508640000**
**H**	**5.4406430000**	**-4.0906110000**	**-0.2127480000**
**H**	**4.0313020000**	**-2.2071590000**	**-2.6162510000**
**H**	**3.4179960000**	**-1.2166310000**	**-1.2580850000**
**H**	**5.9504530000**	**-2.4193450000**	**1.5337200000**
**H**	**4.3435960000**	**-3.1287050000**	**1.7526850000**
**H**	**3.1678620000**	**3.7959470000**	**-1.1377640000**
**H**	**2.6591210000**	**4.4783640000**	**-2.6974200000**
**N**	**-4.2423910000**	**-0.5147170000**	**-0.4813460000**
**H**	**-4.1143770000**	**-0.6039650000**	**0.5198400000**
**H**	**-4.1717290000**	**-1.4416600000**	**-0.8830960000**
**H**	**-3.4212700000**	**1.3809460000**	**-0.6541600000**
**H**	**-3.3238540000**	**0.3978410000**	**-2.1107940000**
**H**	**-1.0450200000**	**1.9978950000**	**-1.0195290000**
**O**	**-1.6814400000**	**0.0476170000**	**0.7622480000**
**H**	**-0.9991130000**	**-0.5777260000**	**1.0428930000**
**H**	**-1.5768640000**	**-1.0145150000**	**-1.0090940000**
**O**	**-2.8875210000**	**1.7265710000**	**2.5972480000**
**H**	**-2.8520520000**	**1.0437120000**	**3.3050680000**
**H**	**-2.4071450000**	**1.2875800000**	**1.8689950000**
**O**	**-2.6092010000**	**-0.3568470000**	**4.4320650000**
**H**	**-2.6209840000**	**-1.1017810000**	**3.7979950000**
**H**	**-1.6762490000**	**-0.2793880000**	**4.6755570000**
**O**	**-2.5532500000**	**-2.2348790000**	**2.2844340000**
**H**	**-1.6136910000**	**-2.4727450000**	**2.3467620000**

**H**	**-2.5356880000**	**-1.4095190000**	**1.7639440000**
**O**	**-2.8883300000**	**-3.4915010000**	**-0.2520070000**
**H**	**-2.4945450000**	**-4.3727000000**	**-0.1906110000**
**H**	**-2.8784860000**	**-3.1575370000**	**0.6680070000**
**O**	**-5.0928360000**	**-1.5538790000**	**-3.4203620000**
**H**	**-5.6194480000**	**-0.8327160000**	**-3.0292790000**
**H**	**-5.1849460000**	**-2.2760200000**	**-2.7646580000**
**O**	**-5.4414000000**	**3.0782250000**	**-0.8566570000**
**H**	**-5.4135020000**	**2.7350860000**	**0.0600280000**
**H**	**-5.7936820000**	**2.3070650000**	**-1.3421330000**
**O**	**-5.4476330000**	**1.6726150000**	**1.5728610000**
**H**	**-5.4942710000**	**0.8830410000**	**1.0152140000**
**H**	**-4.5263230000**	**1.6645900000**	**1.9275030000**
**O**	**-6.3097730000**	**0.5760370000**	**-1.9098280000**
**H**	**-5.6827150000**	**0.1731640000**	**-1.2457890000**
**H**	**-7.1902450000**	**0.3801120000**	**-1.5605450000**
**O**	**-5.3701120000**	**-3.5495730000**	**-1.4431220000**
**H**	**-5.9486700000**	**-3.0811900000**	**-0.8262830000**
**H**	**-4.5068180000**	**-3.5877440000**	**-0.9719220000**

CR/DAPH$^+$ - S2

**N**	**-0.7487180000**	**-0.0831320000**	**0.1462550000**
**C**	**2.3356870000**	**-0.7191210000**	**-0.3797640000**
**C**	**1.5495080000**	**0.5935670000**	**-0.4909860000**
**C**	**0.4297210000**	**0.7312230000**	**0.5299050000**
**H**	**0.7687090000**	**0.4004830000**	**1.5099190000**
**H**	**-0.4851350000**	**-1.0308660000**	**-0.1591840000**
**H**	**-1.2822030000**	**0.3770540000**	**-0.6033120000**
**H**	**-1.3823050000**	**-0.2081360000**	**0.9505270000**

**O**	**-0.8736300000**	**-1.2105070000**	**-2.5752580000**
**O**	**-0.3071210000**	**-2.9421420000**	**-0.4809450000**
**O**	**-1.0438520000**	**1.5699390000**	**-2.6270770000**
**O**	**0.4439420000**	**-2.0540220000**	**2.1865460000**
**O**	**-1.6972090000**	**2.9589560000**	**-0.3167330000**
**O**	**-2.0192770000**	**-0.6031770000**	**2.6399480000**
**O**	**-3.3166540000**	**0.7233790000**	**-0.6190140000**
**O**	**-3.9162650000**	**-1.6392270000**	**0.6250280000**
**C**	**-1.3489030000**	**-3.2375560000**	**-1.3930250000**
**C**	**-1.0451540000**	**-2.6048850000**	**-2.7290970000**
**C**	**0.7494730000**	**-3.3202860000**	**1.6210780000**
**C**	**-0.9972720000**	**-0.5037770000**	**-3.7945170000**
**C**	**-0.3330380000**	**-3.7720260000**	**0.6682130000**
**C**	**-3.2234220000**	**-1.3119870000**	**2.8874600000**
**C**	**-4.2375250000**	**-0.9723530000**	**1.8293970000**
**C**	**-0.3927140000**	**0.8713010000**	**-3.6602410000**
**C**	**-4.7187350000**	**-1.2272090000**	**-0.4682080000**
**C**	**-3.9213380000**	**-0.3050390000**	**-1.3750450000**
**C**	**-0.4810970000**	**2.8445090000**	**-2.3706200000**
**C**	**-3.0412950000**	**2.9474830000**	**0.1112660000**
**C**	**-1.0102620000**	**-0.8170620000**	**3.6134030000**
**C**	**-0.2639090000**	**-2.1179980000**	**3.4093260000**
**C**	**-3.9040050000**	**1.9996980000**	**-0.7027170000**
**C**	**-1.4552590000**	**3.6383530000**	**-1.5336520000**
**H**	**-1.4276740000**	**-4.3226230000**	**-1.5397880000**
**H**	**-2.3012230000**	**-2.8751490000**	**-0.9847840000**
**H**	**-0.1373800000**	**-3.0458800000**	**-3.1603470000**
**H**	**-1.8858330000**	**-2.8199640000**	**-3.4040320000**
**H**	**0.8885220000**	**-4.0711940000**	**2.4070620000**
**H**	**1.6986940000**	**-3.2113970000**	**1.0918430000**

**H**	**-0.1524570000**	**-4.8140270000**	**0.3739650000**
**H**	**-1.3208150000**	**-3.7148510000**	**1.1477430000**
**H**	**-5.0274040000**	**-2.1180200000**	**-1.0244090000**
**H**	**-5.6282230000**	**-0.7318720000**	**-0.1081700000**
**H**	**-3.1043240000**	**-0.8589170000**	**-1.8432000000**
**H**	**-4.5577950000**	**0.1023520000**	**-2.1700410000**
**H**	**-3.6187530000**	**-1.0206360000**	**3.8690830000**
**H**	**-3.0531180000**	**-2.3952990000**	**2.8896280000**
**H**	**-1.4462120000**	**-0.7962130000**	**4.6198960000**
**H**	**-0.3200190000**	**0.0247640000**	**3.5304260000**
**H**	**-2.3829190000**	**3.7707440000**	**-2.1007100000**
**H**	**-1.0365680000**	**4.6311510000**	**-1.3297580000**
**H**	**-4.2469210000**	**0.1136510000**	**1.6804180000**
**H**	**-5.2291770000**	**-1.2877220000**	**2.1820480000**
**H**	**-3.4775080000**	**3.9546140000**	**0.0887670000**
**H**	**-3.0173760000**	**2.6121560000**	**1.1507050000**
**H**	**-3.9620240000**	**2.3160340000**	**-1.7522280000**
**H**	**-4.9237860000**	**2.0014070000**	**-0.2908420000**
**H**	**0.6838760000**	**0.7838000000**	**-3.4550650000**
**H**	**-0.5059930000**	**1.3996360000**	**-4.6177630000**
**H**	**-0.3041540000**	**3.3843770000**	**-3.3110150000**
**H**	**0.4800070000**	**2.7416070000**	**-1.8485450000**
**H**	**-0.4721630000**	**-1.0320440000**	**-4.6010520000**
**H**	**-2.0586030000**	**-0.4252110000**	**-4.0691920000**
**H**	**-0.9697570000**	**-2.9581610000**	**3.4017530000**
**H**	**0.4310220000**	**-2.2756110000**	**4.2445540000**
**N**	**2.9990350000**	**-0.9769530000**	**0.8827190000**
**H**	**2.3259370000**	**-1.0922600000**	**1.6319570000**
**H**	**3.6149910000**	**-0.2122650000**	**1.1292550000**
**H**	**1.6644040000**	**-1.5517980000**	**-0.6124050000**

**H**	**3.0904450000**	**-0.7205650000**	**-1.1760380000**
**H**	**0.1012360000**	**1.7671830000**	**0.5843360000**
**O**	**2.3709930000**	**1.7268820000**	**-0.2750230000**
**H**	**3.1285240000**	**1.6723000000**	**-0.8606940000**
**H**	**1.0970140000**	**0.6262610000**	**-1.4896950000**
**O**	**3.0174660000**	**2.1292320000**	**2.3629250000**
**H**	**2.9541400000**	**1.2492390000**	**2.7632040000**
**H**	**2.7559580000**	**1.9830230000**	**1.4236260000**
**O**	**4.2393670000**	**-3.2041940000**	**-0.1755600000**
**H**	**3.8350680000**	**-2.4730140000**	**0.3621790000**
**H**	**3.4983120000**	**-3.4842630000**	**-0.7551730000**
**O**	**5.6212790000**	**1.6369670000**	**1.2316930000**
**H**	**5.7506620000**	**0.6898180000**	**1.3795030000**
**H**	**4.7919300000**	**1.8374380000**	**1.7098320000**
**O**	**4.7942900000**	**1.4545530000**	**-1.4281080000**
**H**	**5.1354330000**	**1.6090310000**	**-0.5186970000**
**H**	**4.9741390000**	**0.4978920000**	**-1.5531520000**
**O**	**5.7002450000**	**-1.2099270000**	**-1.4402750000**
**H**	**5.1528410000**	**-1.9442220000**	**-1.0729860000**
**H**	**6.2762880000**	**-0.9718770000**	**-0.7008340000**
**O**	**0.4359760000**	**2.7188720000**	**3.3851550000**
**H**	**1.3271750000**	**2.5500190000**	**3.0118070000**
**H**	**-0.1242180000**	**2.0547880000**	**2.9600920000**
**O**	**-0.4088890000**	**4.5981630000**	**1.5276400000**
**H**	**-0.0821090000**	**4.0141480000**	**2.2465690000**
**H**	**-0.8878000000**	**3.9794160000**	**0.9406950000**
**O**	**2.0290940000**	**-4.0644190000**	**-1.7003180000**
**H**	**1.9296100000**	**-4.9558560000**	**-1.3375590000**
**H**	**1.2748160000**	**-3.5803000000**	**-1.3119440000**
**O**	**1.7226420000**	**4.5145400000**	**-0.2579780000**

**H**	**1.9430970000**	**3.5666530000**	**-0.2639180000**
**H**	**1.0049420000**	**4.5778090000**	**0.4081220000**

CR/CsF/DAPH$^+$ - S1

**O**	**-0.4661690000**	**-2.5262480000**	**1.0503990000**
**O**	**-0.2298730000**	**-0.3092120000**	**2.7964320000**
**O**	**-2.0238970000**	**-3.0502280000**	**-1.2540240000**
**O**	**0.1229910000**	**2.2586870000**	**1.6342700000**
**O**	**-4.0320440000**	**-1.1501480000**	**-1.6196720000**
**O**	**-0.8769240000**	**3.3319680000**	**-0.6546070000**
**O**	**-3.7388530000**	**-0.3302970000**	**1.2342910000**
**O**	**-3.3559740000**	**2.4496690000**	**0.5245270000**
**C**	**-1.0300790000**	**-1.4340990000**	**3.0955110000**
**C**	**-0.4327640000**	**-2.6638200000**	**2.4619110000**
**C**	**0.1377370000**	**2.0430230000**	**3.0329500000**
**C**	**-0.4274210000**	**-3.7597150000**	**0.3447450000**
**C**	**-0.7644660000**	**0.8779170000**	**3.3483030000**
**C**	**-1.8877060000**	**4.1887400000**	**-0.1642480000**
**C**	**-3.2283590000**	**3.5299120000**	**-0.3774870000**
**C**	**-0.6907700000**	**-3.4869090000**	**-1.1135590000**
**C**	**-4.6097520000**	**1.8058220000**	**0.4497350000**
**C**	**-4.6550700000**	**0.7161670000**	**1.5028530000**
**C**	**-2.3352490000**	**-2.6127130000**	**-2.5600670000**
**C**	**-4.9517710000**	**-1.4727040000**	**-0.5935000000**
**C**	**0.4394690000**	**3.6508300000**	**-0.2595130000**
**C**	**0.6774250000**	**3.4924160000**	**1.2362920000**
**C**	**-4.2922810000**	**-1.5522830000**	**0.7724750000**
**C**	**-3.7798220000**	**-2.1625090000**	**-2.5819870000**
**H**	**-1.0821800000**	**-1.5876810000**	**4.1829060000**

**H**	**-2.0489220000**	**-1.2764810000**	**2.7158440000**
**H**	**0.6000180000**	**-2.8163260000**	**2.8012100000**
**H**	**-1.0270250000**	**-3.5333230000**	**2.7679820000**
**H**	**-0.2462180000**	**2.9304380000**	**3.5532190000**
**H**	**1.1619150000**	**1.8470400000**	**3.3736510000**
**H**	**-0.8550210000**	**0.7827340000**	**4.4391070000**
**H**	**-1.7642140000**	**1.0673820000**	**2.9313490000**
**H**	**-5.4201460000**	**2.5239560000**	**0.6429010000**
**H**	**-4.7581320000**	**1.3813140000**	**-0.5535370000**
**H**	**-4.3784210000**	**1.1513880000**	**2.4674920000**
**H**	**-5.6774950000**	**0.3300640000**	**1.5877710000**
**H**	**-1.8594420000**	**5.1513090000**	**-0.6921230000**
**H**	**-1.7669730000**	**4.3738470000**	**0.9087880000**
**H**	**0.7137410000**	**4.6699130000**	**-0.5639740000**
**H**	**1.0782610000**	**2.9559880000**	**-0.8113050000**
**H**	**-4.4260410000**	**-3.0264910000**	**-2.3960710000**
**H**	**-4.0146190000**	**-1.7712160000**	**-3.5770600000**
**H**	**-3.3112750000**	**3.1776170000**	**-1.4161050000**
**H**	**-4.0232580000**	**4.2680860000**	**-0.2031990000**
**H**	**-5.4279100000**	**-2.4401410000**	**-0.7882010000**
**H**	**-5.7479880000**	**-0.7201310000**	**-0.5979100000**
**H**	**-3.4524640000**	**-2.2489020000**	**0.7176700000**
**H**	**-5.0254360000**	**-1.9307710000**	**1.4987710000**
**H**	**0.0028380000**	**-2.7199920000**	**-1.4806270000**
**H**	**-0.5194060000**	**-4.4086940000**	**-1.6874900000**
**H**	**-2.2159890000**	**-3.4306530000**	**-3.2859600000**
**H**	**-1.6550060000**	**-1.8074360000**	**-2.8657770000**
**H**	**0.5547710000**	**-4.2335530000**	**0.4618880000**
**H**	**-1.1983370000**	**-4.4331940000**	**0.7379670000**
**H**	**0.2121120000**	**4.3084430000**	**1.8051920000**

**H**	**1.7573070000**	**3.5237650000**	**1.4360940000**
**F**	**0.4711150000**	**-0.7511440000**	**-2.7998880000**
**Cs**	**-1.2099540000**	**0.2161480000**	**-0.4100830000**
**N**	**2.6534180000**	**-1.9562980000**	**-2.6705180000**
**C**	**3.3873080000**	**-0.0122640000**	**0.5369910000**
**C**	**2.6335650000**	**-0.8185470000**	**-0.5174440000**
**C**	**3.4792880000**	**-1.1838190000**	**-1.7240760000**
**H**	**3.8174680000**	**-0.2727840000**	**-2.2221590000**
**H**	**3.1586010000**	**-2.1368550000**	**-3.5308780000**
**H**	**1.3451490000**	**-1.2472990000**	**-2.8438350000**
**H**	**2.4419830000**	**-2.8538570000**	**-2.2445090000**
**N**	**2.5597150000**	**0.4235850000**	**1.6466930000**
**H**	**2.2227910000**	**-0.3613470000**	**2.1907350000**
**H**	**1.7338310000**	**0.9133540000**	**1.3172330000**
**H**	**4.2127640000**	**-0.6157740000**	**0.9250880000**
**H**	**3.8299990000**	**0.8689600000**	**0.0582430000**
**H**	**4.3612510000**	**-1.7495260000**	**-1.4035610000**
**O**	**2.1350890000**	**-2.0378360000**	**0.0080530000**
**H**	**1.3210410000**	**-1.8857700000**	**0.5023320000**
**H**	**1.8011070000**	**-0.2048510000**	**-0.8866180000**
**O**	**3.5107710000**	**-2.7197090000**	**2.4713800000**
**H**	**2.7698860000**	**-2.7923770000**	**3.0881970000**
**H**	**3.0849180000**	**-2.5205720000**	**1.6127710000**
**O**	**0.5445040000**	**1.9079270000**	**-3.4430390000**
**H**	**-0.0220800000**	**2.3249200000**	**-2.7766690000**
**H**	**0.5347190000**	**0.9561850000**	**-3.2174730000**
**O**	**3.0166340000**	**2.4832660000**	**-2.3941230000**
**H**	**3.0039060000**	**1.9304720000**	**-1.6019000000**
**H**	**2.1496810000**	**2.2856050000**	**-2.8222450000**
**O**	**4.6215700000**	**-0.3176700000**	**3.5017350000**

H	4.0026430000	0.2891080000	3.0481930000
H	4.2991800000	-1.1952430000	3.1999880000
O	6.7749800000	-0.4824700000	1.7110470000
H	6.6236580000	0.1172200000	0.9637750000
H	6.0225190000	-0.3241400000	2.3194220000
O	5.2168860000	1.2876520000	-3.6341950000
H	4.4261810000	1.7267620000	-3.2556360000
H	5.7774280000	1.1433740000	-2.8531130000
O	6.4110570000	0.8067850000	-0.9782930000
H	5.8966650000	-0.0126700000	-1.0226940000
H	5.7594760000	1.4702150000	-0.7075630000

CR/CSF/DAPH$^+$ - S2

O	-0.1396870000	-3.1027920000	0.3275660000
O	-1.5352440000	-1.7130630000	-1.5456060000
O	2.2126310000	-2.5994270000	1.8845950000
O	-1.2019200000	1.0189790000	-1.2627690000
O	4.0247480000	-0.3893540000	1.2618670000
O	0.9494540000	2.5882710000	-0.4054640000
O	4.4339690000	-0.7108600000	-1.6043390000
O	3.0319480000	1.7723450000	-2.1429300000
C	-1.0255610000	-2.9840520000	-1.8738010000
C	-0.9319280000	-3.8049550000	-0.6089390000
C	-2.1735770000	0.4916710000	-2.1491430000
C	0.2463180000	-3.8823360000	1.4468510000
C	-1.6844320000	-0.8463120000	-2.6475570000
C	1.2690870000	3.2823950000	-1.5880450000
C	2.7465570000	3.1261840000	-1.8532340000
C	0.9857830000	-3.0190800000	2.4387460000

**C**	**4.3694410000**	**1.5663240000**	**-2.5394350000**
**C**	**4.6007860000**	**0.0841190000**	**-2.7597370000**
**C**	**2.9878760000**	**-1.8595630000**	**2.8143330000**
**C**	**5.1498260000**	**0.1173560000**	**0.5790500000**
**C**	**-0.3545650000**	**2.7873730000**	**0.0951850000**
**C**	**-1.4577650000**	**2.3483730000**	**-0.8553870000**
**C**	**5.4973050000**	**-0.6879400000**	**-0.6656150000**
**C**	**4.2857300000**	**-1.4409220000**	**2.1690830000**
**H**	**-1.6768600000**	**-3.4985190000**	**-2.5928240000**
**H**	**-0.0284570000**	**-2.8865460000**	**-2.3319760000**
**H**	**-1.9341490000**	**-3.9852020000**	**-0.1973870000**
**H**	**-0.4790570000**	**-4.7754860000**	**-0.8496110000**
**H**	**-2.3078510000**	**1.1615000000**	**-3.0092380000**
**H**	**-3.1321320000**	**0.3846780000**	**-1.6280960000**
**H**	**-2.4053840000**	**-1.2524650000**	**-3.3695040000**
**H**	**-0.7195930000**	**-0.7223610000**	**-3.1642820000**
**H**	**4.5779440000**	**2.0974940000**	**-3.4799070000**
**H**	**5.0599540000**	**1.9609000000**	**-1.7797840000**
**H**	**3.8687060000**	**-0.2835510000**	**-3.4830850000**
**H**	**5.6004730000**	**-0.0611680000**	**-3.1897300000**
**H**	**1.0362450000**	**4.3521540000**	**-1.4874590000**
**H**	**0.7077030000**	**2.8825090000**	**-2.4433750000**
**H**	**-0.5167500000**	**3.8437250000**	**0.3523540000**
**H**	**-0.3881000000**	**2.2009320000**	**1.0159790000**
**H**	**4.7334230000**	**-2.3054800000**	**1.6619200000**
**H**	**4.9782460000**	**-1.0980730000**	**2.9499270000**
**H**	**3.3162000000**	**3.4536280000**	**-0.9729320000**
**H**	**3.0235390000**	**3.7642170000**	**-2.7040950000**
**H**	**6.0245790000**	**0.1550680000**	**1.2435190000**
**H**	**4.8912070000**	**1.1425330000**	**0.3042180000**

**H**	**5.7042270000**	**-1.7275320000**	**-0.3955030000**
**H**	**6.4087660000**	**-0.2771130000**	**-1.1183960000**
**H**	**0.3821940000**	**-2.1459770000**	**2.7184410000**
**H**	**1.1592180000**	**-3.6163420000**	**3.3458030000**
**H**	**3.2118740000**	**-2.4833510000**	**3.6908180000**
**H**	**2.4336890000**	**-0.9712690000**	**3.1417990000**
**H**	**-0.6417100000**	**-4.3059840000**	**1.9364910000**
**H**	**0.8893860000**	**-4.7089000000**	**1.1192080000**
**H**	**-1.5132240000**	**3.0019680000**	**-1.7367800000**
**H**	**-2.4216720000**	**2.4106540000**	**-0.3323110000**
**F**	**0.3720630000**	**0.3114530000**	**2.4937470000**
**Cs**	**1.4895400000**	**-0.4756190000**	**-0.4386550000**
**N**	**-1.6657020000**	**-0.5732040000**	**1.3555310000**
**C**	**-4.2478220000**	**-1.9630570000**	**0.5305510000**
**C**	**-4.1667200000**	**-0.6946840000**	**1.3793740000**
**C**	**-2.8731390000**	**-0.5885790000**	**2.1847490000**
**H**	**-2.8908850000**	**0.3314280000**	**2.7772940000**
**H**	**-1.4486350000**	**-1.4945250000**	**0.9807500000**
**H**	**-0.4660020000**	**-0.0425180000**	**2.0927600000**
**H**	**-1.8101610000**	**0.0168860000**	**0.5372910000**
**N**	**-5.4799550000**	**-2.1463270000**	**-0.2132720000**
**H**	**-5.6681220000**	**-1.3095900000**	**-0.7569130000**
**H**	**-6.2676010000**	**-2.2672830000**	**0.4131070000**
**H**	**-3.4202060000**	**-1.9452540000**	**-0.1847360000**
**H**	**-4.0955900000**	**-2.8289160000**	**1.1834370000**
**H**	**-2.8035140000**	**-1.4177640000**	**2.8945770000**
**O**	**-4.2673330000**	**0.4140000000**	**0.4780950000**
**H**	**-4.6530220000**	**1.1571670000**	**0.9397170000**
**H**	**-5.0168320000**	**-0.6805600000**	**2.0769320000**
**O**	**-5.7529770000**	**1.1041570000**	**-1.7241510000**

**H**	**-5.2518930000**	**0.7152740000**	**-0.9746530000**
**H**	**-5.2153840000**	**0.8865040000**	**-2.4988310000**
**O**	**-4.7974720000**	**3.6228300000**	**-0.9886950000**
**H**	**-5.1556030000**	**2.7623750000**	**-1.3047820000**
**H**	**-3.9225350000**	**3.6745260000**	**-1.3964370000**
**O**	**-4.3291890000**	**-3.7197030000**	**-2.2134620000**
**H**	**-3.8992410000**	**-2.9752250000**	**-2.6544650000**
**H**	**-4.8504000000**	**-3.2774910000**	**-1.4973930000**
**O**	**2.2341400000**	**2.3333330000**	**2.0365080000**
**H**	**2.0116690000**	**2.5144720000**	**1.1015730000**
**H**	**1.6126480000**	**1.6158300000**	**2.2591170000**
**O**	**-4.5876170000**	**2.8271410000**	**1.6661900000**
**H**	**-4.5597340000**	**3.2063270000**	**0.7584800000**
**H**	**-3.6558570000**	**2.7826170000**	**1.9554250000**
**O**	**0.4444020000**	**4.3605540000**	**2.8970160000**
**H**	**-0.4050460000**	**3.8830480000**	**2.8972300000**
**H**	**1.0887740000**	**3.6757940000**	**2.6265280000**
**O**	**-1.9641730000**	**2.7760630000**	**2.9401010000**
**H**	**-1.4340720000**	**1.9658250000**	**2.9867910000**
**H**	**-2.3036930000**	**2.8834160000**	**3.8411310000**

CR/CsF/DAPH$^+$ - S3

**O**	**1.7498380000**	**0.2174070000**	**2.8593830000**
**O**	**1.4838040000**	**-2.4421850000**	**2.0564890000**
**O**	**2.2430170000**	**2.6718010000**	**1.6705480000**
**O**	**-0.4005240000**	**-2.7957660000**	**0.0245380000**
**O**	**3.2966700000**	**2.5041740000**	**-0.9533100000**
**O**	**-0.2744620000**	**-1.6090950000**	**-2.4303850000**
**O**	**4.0305050000**	**-0.3394410000**	**-0.1960810000**

**O**	**2.5956700000**	**-1.8079660000**	**-2.2324620000**
**C**	**2.7109710000**	**-1.9199320000**	**2.5314280000**
**C**	**2.4226530000**	**-0.8242230000**	**3.5249350000**
**C**	**0.3005300000**	**-3.8874920000**	**0.5925080000**
**C**	**1.6867850000**	**1.4251710000**	**3.5940860000**
**C**	**1.6568460000**	**-3.4065610000**	**1.0415710000**
**C**	**0.5049500000**	**-2.5884390000**	**-3.0781540000**
**C**	**1.8462740000**	**-1.9884610000**	**-3.4201560000**
**C**	**1.2481160000**	**2.5322130000**	**2.6697420000**
**C**	**3.8526420000**	**-1.2092230000**	**-2.4681020000**
**C**	**4.6387390000**	**-1.1635110000**	**-1.1737950000**
**C**	**1.9538130000**	**3.6541310000**	**0.7075430000**
**C**	**4.5997430000**	**1.9530390000**	**-0.8960340000**
**C**	**-1.5298010000**	**-2.0421100000**	**-1.9468620000**
**C**	**-1.4301420000**	**-3.1381600000**	**-0.8941290000**
**C**	**4.7203650000**	**0.8540290000**	**0.1498740000**
**C**	**3.1156960000**	**3.7298450000**	**-0.2565070000**
**H**	**3.2974970000**	**-2.7106110000**	**3.0193710000**
**H**	**3.2933230000**	**-1.5097310000**	**1.6952210000**
**H**	**1.8192020000**	**-1.2102760000**	**4.3606800000**
**H**	**3.3761790000**	**-0.4680210000**	**3.9385860000**
**H**	**0.4386530000**	**-4.6788060000**	**-0.1541420000**
**H**	**-0.2585390000**	**-4.3085200000**	**1.4386840000**
**H**	**2.2377540000**	**-4.2617810000**	**1.4146180000**
**H**	**2.1891250000**	**-2.9711380000**	**0.1829200000**
**H**	**4.4219150000**	**-1.7948790000**	**-3.2044580000**
**H**	**3.7155390000**	**-0.1959790000**	**-2.8730300000**
**H**	**4.6997130000**	**-2.1762690000**	**-0.7622620000**
**H**	**5.6590060000**	**-0.8262630000**	**-1.3904720000**
**H**	**0.0110590000**	**-2.9287940000**	**-3.9983420000**

H	0.6718620000	-3.4591300000	-2.4314470000
H	-2.1695700000	-2.3972570000	-2.7662450000
H	-2.0021650000	-1.1648660000	-1.5059950000
H	4.0172570000	3.9797660000	0.3124300000
H	2.9331770000	4.5305680000	-0.9813410000
H	1.6991250000	-1.0251910000	-3.9285440000
H	2.3805790000	-2.6606650000	-4.1050660000
H	5.3393330000	2.7316600000	-0.6760530000
H	4.8346440000	1.5646480000	-1.8914720000
H	4.2615500000	1.1977430000	1.0802810000
H	5.7801210000	0.6334540000	0.3341170000
H	0.2741160000	2.2863340000	2.2262590000
H	1.1499400000	3.4617620000	3.2470800000
H	1.8213640000	4.6404630000	1.1751170000
H	1.0173190000	3.4215240000	0.1770390000
H	0.9761510000	1.3376760000	4.4270760000
H	2.6774890000	1.6607150000	4.0056760000
H	-1.1970750000	-4.1069520000	-1.3501990000
H	-2.3961620000	-3.2215340000	-0.3852590000
F	-1.2870540000	0.9908450000	1.4693930000
Cs	1.0170750000	0.2163620000	-0.2656620000
N	-1.1816090000	-1.2436550000	2.3931180000
C	-4.8845270000	-1.6865950000	1.7185470000
C	-3.5709370000	-0.9214660000	1.8532700000
C	-2.5545650000	-1.7100340000	2.6944020000
H	-2.7552240000	-1.5700520000	3.7575760000
H	-0.5069620000	-1.4608160000	3.1190960000
H	-1.1717420000	-0.1260950000	2.0969720000
H	-0.8526990000	-1.7305570000	1.5511870000
N	-5.9257390000	-0.9925910000	0.9889740000

**H**	**-5.5417730000**	**-0.6374030000**	**0.1208050000**
**H**	**-6.2610710000**	**-0.1942260000**	**1.5148950000**
**H**	**-4.6690670000**	**-2.6314660000**	**1.2028670000**
**H**	**-5.2579800000**	**-1.9516060000**	**2.7133260000**
**H**	**-2.6176420000**	**-2.7797860000**	**2.4722740000**
**O**	**-3.0644650000**	**-0.6785750000**	**0.5549950000**
**H**	**-2.4873100000**	**0.1051890000**	**0.6472140000**
**H**	**-3.7581870000**	**0.0403490000**	**2.3474240000**
**O**	**-3.5510380000**	**1.0174500000**	**-1.6709460000**
**H**	**-2.9924520000**	**1.6818030000**	**-1.1903390000**
**H**	**-3.5915940000**	**0.2680520000**	**-1.0464920000**
**O**	**-1.9083240000**	**2.7807220000**	**-0.3622780000**
**H**	**-1.1589280000**	**2.7892440000**	**-1.0040860000**
**H**	**-1.6348950000**	**2.1878230000**	**0.3741880000**
**O**	**-1.3854190000**	**0.7417330000**	**-3.5187130000**
**H**	**-2.1646970000**	**0.7397860000**	**-2.9232110000**
**H**	**-0.8809550000**	**-0.0551450000**	**-3.2607200000**
**O**	**-5.9517100000**	**2.0222500000**	**-0.7219470000**
**H**	**-5.8093550000**	**1.8183770000**	**0.2144110000**
**H**	**-5.1573150000**	**1.6346170000**	**-1.1595270000**
**O**	**-7.9410360000**	**-0.0327670000**	**-0.9673680000**
**H**	**-7.5701850000**	**-0.6559460000**	**-0.3214230000**
**H**	**-7.2993480000**	**0.7037950000**	**-0.9530310000**
**O**	**-4.2467410000**	**4.3441380000**	**-0.3573790000**
**H**	**-4.9124320000**	**3.6535580000**	**-0.5224660000**
**H**	**-3.4084080000**	**3.8452990000**	**-0.3392540000**
**O**	**0.0410510000**	**2.6936270000**	**-2.3043340000**
**H**	**-0.1193490000**	**3.5154570000**	**-2.7886210000**
**H**	**-0.4577050000**	**2.0029300000**	**-2.8126350000**

CR/DAPH⁺ - G1

N	-1.1359820000	0.0190020000	-0.6610140000
C	-4.1873910000	-2.2397540000	-1.1623130000
C	-2.7702790000	-1.8460030000	-0.7430520000
C	-2.4432600000	-0.4279020000	-1.1966290000
H	-2.4001540000	-0.3675270000	-2.2858380000
H	-0.9105010000	0.9662770000	-0.9991120000
H	-0.3888720000	-0.6462770000	-0.9161930000
H	-1.1578670000	0.0862110000	0.3683870000
O	3.0672220000	0.0727980000	1.2537210000
O	1.1989800000	1.8635670000	-0.1908630000
O	3.6096520000	-1.6643020000	-0.9776470000
O	-0.7555280000	2.5449680000	-1.9194830000
O	0.6111580000	-2.0432920000	-1.4787460000
O	-2.5224160000	2.3006110000	0.2998110000
O	-0.0005960000	-1.9499670000	1.3355580000
O	-1.0387010000	0.5983650000	2.1376980000
C	1.8230040000	2.0809940000	1.0616440000
C	3.2056360000	1.4722160000	1.1640300000
C	0.5887580000	2.6143790000	-2.3534620000
C	4.2744700000	-0.6566080000	1.1305330000
C	1.5775940000	2.7640320000	-1.2051060000
C	-2.0810300000	2.6820810000	1.5848410000
C	-2.0992050000	1.4741580000	2.4896920000
C	4.6361110000	-0.9490470000	-0.3182360000
C	-0.9272240000	-0.5143490000	3.0123660000
C	0.2532560000	-1.3578020000	2.5999510000
C	2.7552110000	-0.8590800000	-1.7622020000
C	0.8157040000	-3.1273290000	-0.5785190000
C	-2.5965250000	3.3425860000	-0.6515310000

**C**	**-1.2473360000**	**3.6925190000**	**-1.2484430000**
**C**	**1.1179440000**	**-2.6607920000**	**0.8250200000**
**C**	**1.6693600000**	**-1.7200800000**	**-2.3703460000**
**H**	**1.8844370000**	**3.1592170000**	**1.2655450000**
**H**	**1.1650500000**	**1.6229880000**	**1.8019130000**
**H**	**3.8136920000**	**1.7573720000**	**0.2935500000**
**H**	**3.7079040000**	**1.8633040000**	**2.0602620000**
**H**	**0.7290230000**	**3.4339220000**	**-3.0691970000**
**H**	**0.7649680000**	**1.6732810000**	**-2.8794910000**
**H**	**2.5878780000**	**2.5605070000**	**-1.5867920000**
**H**	**1.5802820000**	**3.7908130000**	**-0.8140230000**
**H**	**-0.7631970000**	**-0.1632740000**	**4.0393980000**
**H**	**-1.8543110000**	**-1.0998190000**	**2.9902130000**
**H**	**1.1603120000**	**-0.7461170000**	**2.5357900000**
**H**	**0.4068660000**	**-2.1393830000**	**3.3560460000**
**H**	**-2.7502400000**	**3.4467020000**	**2.0024430000**
**H**	**-1.0628400000**	**3.0919510000**	**1.5521180000**
**H**	**-3.0450280000**	**4.2428120000**	**-0.2114720000**
**H**	**-3.2586460000**	**2.9822930000**	**-1.4413970000**
**H**	**2.1071040000**	**-2.6353670000**	**-2.7835480000**
**H**	**1.1990830000**	**-1.1716320000**	**-3.1907360000**
**H**	**-3.0608270000**	**0.9539530000**	**2.4008560000**
**H**	**-1.9727530000**	**1.8052330000**	**3.5284330000**
**H**	**1.6393020000**	**-3.7566130000**	**-0.9269140000**
**H**	**-0.1026000000**	**-3.7226700000**	**-0.5858920000**
**H**	**1.9897610000**	**-2.0056310000**	**0.8124460000**
**H**	**1.3285760000**	**-3.5351360000**	**1.4563970000**
**H**	**4.8668600000**	**-0.0242550000**	**-0.8634070000**
**H**	**5.5353370000**	**-1.5710810000**	**-0.3349680000**
**H**	**3.3262370000**	**-0.3970040000**	**-2.5826080000**

H	2.2943320000	-0.0672780000	-1.1584060000
H	5.1030470000	-0.1339190000	1.6273990000
H	4.1121340000	-1.6003350000	1.6563480000
H	-0.5561510000	4.0054460000	-0.4579370000
H	-1.3605060000	4.5249660000	-1.9546510000
N	-4.5815970000	-3.5842510000	-0.7982590000
H	-4.4178050000	-3.7280210000	0.1911420000
H	-4.0366740000	-4.2734480000	-1.3024310000
H	-4.8828420000	-1.5360620000	-0.6908560000
H	-4.2942450000	-2.1157930000	-2.2449450000
H	-3.1910490000	0.2736240000	-0.8262910000
O	-2.6904410000	-1.9406130000	0.6663400000
H	-1.7785590000	-2.1488590000	0.9121940000
H	-2.0568970000	-2.5410330000	-1.2093990000

CR/DAPH$^+$ - G2

N	0.0979200000	-0.2621610000	-0.4475120000
C	0.8553110000	-3.3559340000	-0.2279740000
C	-0.3285040000	-2.6593200000	-0.9117760000
C	0.0128710000	-1.3002310000	-1.5032040000
H	0.9704760000	-1.3414980000	-2.0190030000
H	0.6335670000	-0.5822940000	0.3719780000
H	-0.8412680000	0.0283290000	-0.1445540000
H	0.5868370000	0.5753250000	-0.7989130000
O	-0.9168580000	-0.8891680000	2.2488430000
O	1.8341550000	-0.8056990000	1.8875490000
O	-2.9886950000	-0.7962780000	0.3883590000
O	3.1168000000	-0.7893250000	-0.7246660000
O	-2.5977360000	0.4556840000	-2.0557680000

O	1.8549640000	1.6717020000	-1.5772080000
O	-1.5283040000	1.9679830000	0.0132230000
O	0.8696340000	2.9420380000	0.9021520000
C	1.2247200000	-0.0585540000	2.9242630000
C	-0.0507490000	-0.7402320000	3.3556600000
C	3.7187990000	-1.2171780000	0.4882370000
C	-2.2581480000	-1.1294450000	2.6290470000
C	3.1860370000	-0.4394290000	1.6697080000
C	2.2760870000	2.9106470000	-1.0282910000
C	1.1186900000	3.5772450000	-0.3359570000
C	-3.0323500000	-1.6988030000	1.4668070000
C	-0.3186950000	3.3942850000	1.5284370000
C	-1.4149850000	2.3547430000	1.3669010000
C	-3.6184970000	-1.2889990000	-0.7810820000
C	-2.5886450000	1.8658120000	-2.0893350000
C	2.8647920000	0.9835530000	-2.2970280000
C	3.8232450000	0.2361650000	-1.3947380000
C	-2.6259590000	2.4820170000	-0.7022860000
C	-3.8410360000	-0.1340570000	-1.7278920000
H	1.8923020000	-0.0027620000	3.7939410000
H	1.0338280000	0.9628750000	2.5704810000
H	0.1721570000	-1.7238690000	3.7884230000
H	-0.5189230000	-0.1205220000	4.1336790000
H	4.8084970000	-1.1134100000	0.4370960000
H	3.4898490000	-2.2792140000	0.6006700000
H	3.7798410000	-0.6748170000	2.5627950000
H	3.2550450000	0.6423800000	1.4852380000
H	-0.1100890000	3.5509040000	2.5915130000
H	-0.6281870000	4.3583440000	1.1077700000
H	-1.1542810000	1.4499750000	1.9208790000

H	-2.3678330000	2.7321910000	1.7576350000
H	2.6326060000	3.5614410000	-1.8370680000
H	3.0932320000	2.7696420000	-0.3106820000
H	3.4239830000	1.6857420000	-2.9277060000
H	2.3488050000	0.2812800000	-2.9547380000
H	-4.5062440000	0.5925850000	-1.2491880000
H	-4.3329790000	-0.4978290000	-2.6378510000
H	0.2362920000	3.5223820000	-0.9840720000
H	1.3707270000	4.6342520000	-0.1734480000
H	-3.4202650000	2.2576010000	-2.6894340000
H	-1.6546740000	2.1434450000	-2.5833570000
H	-3.5600900000	2.2346170000	-0.1816570000
H	-2.5689120000	3.5763910000	-0.7955310000
H	-2.6075110000	-2.6714930000	1.1801250000
H	-4.0698940000	-1.8714490000	1.7869820000
H	-4.5920590000	-1.7353500000	-0.5360220000
H	-2.9972080000	-2.0610330000	-1.2552300000
H	-2.3010240000	-1.8504520000	3.4558570000
H	-2.7193180000	-0.1908580000	2.9673010000
H	4.2747970000	0.9277670000	-0.6723860000
H	4.6350930000	-0.1909950000	-1.9978970000
N	2.0159580000	-3.6302370000	-1.0519350000
H	2.4529880000	-2.7705450000	-1.3646100000
H	1.7575120000	-4.1664110000	-1.8711390000
H	1.1603400000	-2.7614260000	0.6391780000
H	0.4888840000	-4.3051060000	0.1825180000
H	-0.7659590000	-0.9904550000	-2.1971060000
O	-0.8277870000	-3.4098770000	-2.0044640000
H	-1.0181170000	-4.2999640000	-1.7031320000
H	-1.1072170000	-2.5091140000	-0.1543310000

CR/CsF/DAPH$^+$ - G1

O	1.3836110000	-1.4171660000	1.7791870000
O	0.3633470000	1.1048780000	2.5649570000
O	0.5602470000	-3.3664030000	-0.1007000000
O	-0.3030900000	2.9587470000	0.5182230000
O	-2.0099410000	-2.8765840000	-1.0655070000
O	-1.3448610000	2.5756550000	-1.9634510000
O	-2.5138200000	-1.0825200000	1.2648750000
O	-3.3112090000	1.1148590000	-0.4422480000
C	0.1133610000	-0.0794610000	3.2926290000
C	1.2769820000	-1.0242770000	3.1381150000
C	-0.3897300000	3.2748780000	1.8950330000
C	2.0646890000	-2.6492480000	1.5817100000
C	-0.7143180000	2.0159490000	2.6568050000
C	-2.6885490000	2.9748930000	-1.7865530000
C	-3.5522440000	1.7417250000	-1.6855420000
C	1.9142950000	-3.0548670000	0.1383530000
C	-4.1240440000	-0.0177620000	-0.2218930000
C	-3.8271290000	-0.5617010000	1.1617390000
C	0.2728940000	-3.6072500000	-1.4621440000
C	-2.8238820000	-3.2104760000	0.0430890000
C	-0.3769690000	3.5835490000	-1.7684230000
C	-0.3051250000	4.0836020000	-0.3317960000
C	-2.3959220000	-2.4955270000	1.3130680000
C	-1.2023480000	-3.9183800000	-1.5918050000
H	-0.0164740000	0.1482640000	4.3603040000
H	-0.8051020000	-0.5555710000	2.9228000000
H	2.2111850000	-0.5515640000	3.4680040000
H	1.0947600000	-1.9026370000	3.7690970000
H	-1.1965720000	4.0003940000	2.0630860000

**H**	**0.5559820000**	**3.7086470000**	**2.2427270000**
**H**	**-0.9047070000**	**2.2745460000**	**3.7075880000**
**H**	**-1.6249190000**	**1.5616830000**	**2.2399300000**
**H**	**-5.1872650000**	**0.2584170000**	**-0.2771470000**
**H**	**-3.9264380000**	**-0.7805640000**	**-0.9886150000**
**H**	**-3.9105970000**	**0.2559660000**	**1.8834960000**
**H**	**-4.5721160000**	**-1.3214850000**	**1.4251070000**
**H**	**-3.0175060000**	**3.5894880000**	**-2.6351100000**
**H**	**-2.8139830000**	**3.5539310000**	**-0.8650190000**
**H**	**-0.5445980000**	**4.4358440000**	**-2.4406880000**
**H**	**0.5742020000**	**3.1223350000**	**-2.0475480000**
**H**	**-1.4161990000**	**-4.8580230000**	**-1.0722240000**
**H**	**-1.4448900000**	**-4.0535230000**	**-2.6507260000**
**H**	**-3.3238600000**	**1.0559090000**	**-2.5145220000**
**H**	**-4.6080220000**	**2.0348850000**	**-1.7663790000**
**H**	**-2.7880530000**	**-4.2875010000**	**0.2412890000**
**H**	**-3.8622420000**	**-2.9681650000**	**-0.2087840000**
**H**	**-1.3355680000**	**-2.6925320000**	**1.4874680000**
**H**	**-2.9779940000**	**-2.8924120000**	**2.1568380000**
**H**	**2.2385390000**	**-2.2351630000**	**-0.5150590000**
**H**	**2.5557970000**	**-3.9256760000**	**-0.0581550000**
**H**	**0.8433900000**	**-4.4703040000**	**-1.8361040000**
**H**	**0.5596840000**	**-2.7412100000**	**-2.0723690000**
**H**	**3.1279840000**	**-2.5389620000**	**1.8270480000**
**H**	**1.6268600000**	**-3.4196040000**	**2.2277480000**
**H**	**-1.1590270000**	**4.7300490000**	**-0.0888340000**
**H**	**0.6085530000**	**4.6796590000**	**-0.2015310000**
**F**	**1.9623760000**	**-0.8706070000**	**-2.4893680000**
**Cs**	**-0.2890640000**	**-0.0336180000**	**-0.5649850000**
**N**	**4.4108570000**	**-0.7951530000**	**-2.0090240000**

**C**	**3.7478730000**	**2.2418270000**	**0.2133510000**
**C**	**3.5862320000**	**0.8697370000**	**-0.4341550000**
**C**	**4.6661590000**	**0.5460380000**	**-1.4515530000**
**H**	**4.6300310000**	**1.2727130000**	**-2.2657910000**
**H**	**5.0584090000**	**-1.0062260000**	**-2.7601550000**
**H**	**2.9624050000**	**-0.8846160000**	**-2.3807840000**
**H**	**4.5631080000**	**-1.4771100000**	**-1.2717590000**
**N**	**2.6668570000**	**2.5984070000**	**1.1140610000**
**H**	**2.6414850000**	**1.9908510000**	**1.9239750000**
**H**	**1.7644510000**	**2.4999210000**	**0.6595960000**
**H**	**4.6923110000**	**2.2684040000**	**0.7648160000**
**H**	**3.8149240000**	**3.0002060000**	**-0.5756050000**
**H**	**5.6530670000**	**0.6054050000**	**-0.9794620000**
**O**	**3.6148470000**	**-0.1743680000**	**0.5265750000**
**H**	**2.7567460000**	**-0.2547450000**	**0.9606040000**
**H**	**2.6289900000**	**0.8554500000**	**-0.9713860000**

CR/CsF/DAPH^{+} - G2

**O**	**-1.3141220000**	**2.2427650000**	**1.0906630000**
**O**	**-2.1807280000**	**-0.2213890000**	**1.8420830000**
**O**	**0.8977230000**	**3.2299240000**	**-0.4404830000**
**O**	**-1.3430970000**	**-2.1885170000**	**0.0859670000**
**O**	**3.1737090000**	**1.5261860000**	**-1.1283770000**
**O**	**0.9659320000**	**-2.4884600000**	**-1.4648150000**
**O**	**3.8352100000**	**0.3093180000**	**1.4343560000**
**O**	**3.0282010000**	**-2.3054380000**	**0.4680520000**
**C**	**-1.9010320000**	**0.7385710000**	**2.8335990000**
**C**	**-2.1183460000**	**2.1132340000**	**2.2457230000**
**C**	**-2.2927210000**	**-2.4724580000**	**1.0985830000**

C	-1.2220270000	3.5714990000	0.6054180000
C	-2.0269390000	-1.5564980000	2.2683070000
C	1.5507840000	-3.6211420000	-0.8668740000
C	2.9877250000	-3.3017120000	-0.5339880000
C	-0.4433310000	3.5896150000	-0.6865500000
C	4.3339760000	-2.0499430000	0.9352050000
C	4.2882970000	-0.9247810000	1.9505400000
C	1.6936920000	3.3215080000	-1.6113000000
C	4.4478000000	1.0026640000	-0.8260700000
C	-0.3204350000	-2.6735370000	-2.0135620000
C	-1.3730870000	-3.0938300000	-0.9993590000
C	4.7673450000	1.0488480000	0.6619450000
C	3.1140500000	2.9290380000	-1.2879010000
H	-2.5554510000	0.6094300000	3.7059080000
H	-0.8588610000	0.6379630000	3.1762370000
H	-3.1766960000	2.2537210000	1.9879030000
H	-1.8439360000	2.8663210000	2.9957260000
H	-2.1913680000	-3.5132370000	1.4346960000
H	-3.3064560000	-2.3209460000	0.7098050000
H	-2.7271780000	-1.7903780000	3.0812670000
H	-1.0041970000	-1.7178470000	2.6441310000
H	4.7504030000	-2.9445220000	1.4214370000
H	4.9979710000	-1.7930840000	0.0969330000
H	3.5850730000	-1.1929470000	2.7429530000
H	5.2812800000	-0.8139900000	2.4056060000
H	1.5262910000	-4.4803090000	-1.5523770000
H	1.0223410000	-3.8964690000	0.0559600000
H	-0.2968360000	-3.4200220000	-2.8202080000
H	-0.5758740000	-1.7053100000	-2.4494940000
H	3.4350290000	3.4497380000	-0.3763360000

H	3.7672450000	3.2395870000	-2.1147850000
H	3.5069010000	-2.9524870000	-1.4368250000
H	3.4818910000	-4.2174980000	-0.1805750000
H	5.2307060000	1.5368380000	-1.3827140000
H	4.4322380000	-0.0321100000	-1.1759320000
H	4.7302590000	2.0807790000	1.0228790000
H	5.7881250000	0.6794190000	0.8234450000
H	-0.8883310000	2.9017210000	-1.4170530000
H	-0.4974470000	4.6066970000	-1.1014810000
H	1.6869800000	4.3547960000	-1.9851800000
H	1.2943460000	2.6588800000	-2.3890340000
H	-2.2267740000	3.9768020000	0.4209410000
H	-0.7238350000	4.2047430000	1.3503340000
H	-1.1959030000	-4.1151940000	-0.6352310000
H	-2.3585200000	-3.0787170000	-1.4846410000
F	-0.3821390000	0.7990320000	-2.6052710000
Cs	0.8835360000	0.0829080000	0.2853270000
N	-2.4115180000	0.4200170000	-1.2023080000
C	-5.1071950000	0.4933510000	0.2129120000
C	-4.8736820000	-0.0430250000	-1.1984490000
C	-3.6844380000	0.6094330000	-1.9022270000
H	-3.5856160000	0.1895320000	-2.9079390000
H	-2.3414410000	1.0059960000	-0.3727590000
H	-1.2220140000	0.6716830000	-2.0888750000
H	-2.3407760000	-0.5360030000	-0.8564720000
N	-6.2584230000	-0.0507100000	0.9097800000
H	-6.2133000000	-1.0629760000	0.8922850000
H	-7.1199970000	0.2140210000	0.4462000000
H	-4.2170620000	0.2788710000	0.8113880000
H	-5.2068870000	1.5835470000	0.1594190000

**H**	**-3.8632880000**	**1.6813630000**	**-2.0256700000**
**O**	**-4.6462310000**	**-1.4502730000**	**-1.0710960000**
**H**	**-4.9233540000**	**-1.8853870000**	**-1.8777240000**
**H**	**-5.7766960000**	**0.1326690000**	**-1.7984840000**

CR/CsF/DAPH^{+} - G3

**O**	**0.6557240000**	**2.1812740000**	**1.6121730000**
**O**	**-0.4379120000**	**-0.1418480000**	**2.7057350000**
**O**	**2.2309650000**	**2.8722720000**	**-0.5607960000**
**O**	**-1.9279120000**	**-1.6280540000**	**0.8684190000**
**O**	**3.5906400000**	**0.6924950000**	**-1.7536840000**
**O**	**-0.9375260000**	**-2.6637340000**	**-1.4547970000**
**O**	**3.0637620000**	**-0.7340090000**	**0.8703820000**
**O**	**1.5676740000**	**-3.0223950000**	**-0.0715970000**
**C**	**0.7910160000**	**0.3954350000**	**3.1588490000**
**C**	**0.7623120000**	**1.8920470000**	**2.9853850000**
**C**	**-1.7971350000**	**-2.0260250000**	**2.2213930000**
**C**	**0.9198350000**	**3.5325180000**	**1.2853580000**
**C**	**-0.4614310000**	**-1.5520290000**	**2.7349720000**
**C**	**-0.4783880000**	**-3.8918940000**	**-0.9381570000**
**C**	**1.0272120000**	**-3.9194140000**	**-1.0245620000**
**C**	**1.0965390000**	**3.6442730000**	**-0.2075400000**
**C**	**2.9776360000**	**-2.9612790000**	**-0.1206160000**
**C**	**3.4824700000**	**-2.0800690000**	**1.0035910000**
**C**	**2.5041040000**	**2.8533690000**	**-1.9394930000**
**C**	**4.5615690000**	**0.1995480000**	**-0.8485570000**
**C**	**-2.3274350000**	**-2.4322340000**	**-1.3504230000**
**C**	**-2.8314870000**	**-2.3982450000**	**0.0863510000**
**C**	**4.0767050000**	**0.2238850000**	**0.5938560000**

**C**	**3.7605650000**	**2.0431040000**	**-2.1631230000**
**H**	**0.9439760000**	**0.1533640000**	**4.2196420000**
**H**	**1.6219930000**	**-0.0232860000**	**2.5750770000**
**H**	**-0.0787440000**	**2.3292080000**	**3.5449440000**
**H**	**1.6908650000**	**2.3094300000**	**3.3987310000**
**H**	**-1.8351260000**	**-3.1192600000**	**2.3001380000**
**H**	**-2.6126930000**	**-1.6102070000**	**2.8278070000**
**H**	**-0.3174290000**	**-1.9208270000**	**3.7602880000**
**H**	**0.3373140000**	**-1.9640240000**	**2.1005740000**
**H**	**3.4072830000**	**-3.9662580000**	**0.0006640000**
**H**	**3.3024620000**	**-2.5685850000**	**-1.0951550000**
**H**	**3.0873200000**	**-2.4577010000**	**1.9522880000**
**H**	**4.5758200000**	**-2.1479840000**	**1.0435240000**
**H**	**-0.8935320000**	**-4.7314720000**	**-1.5125460000**
**H**	**-0.7658520000**	**-4.0159450000**	**0.1136520000**
**H**	**-2.8982630000**	**-3.1883650000**	**-1.9065690000**
**H**	**-2.5078820000**	**-1.4637290000**	**-1.8156130000**
**H**	**4.5746910000**	**2.5109580000**	**-1.5998430000**
**H**	**4.0210840000**	**2.0650250000**	**-3.2268390000**
**H**	**1.3424550000**	**-3.6347480000**	**-2.0381840000**
**H**	**1.3836120000**	**-4.9394380000**	**-0.8272320000**
**H**	**5.4870950000**	**0.7826680000**	**-0.9191930000**
**H**	**4.8060080000**	**-0.8217670000**	**-1.1553810000**
**H**	**3.6231140000**	**1.1966890000**	**0.7993500000**
**H**	**4.9270680000**	**0.0754930000**	**1.2724920000**
**H**	**0.1958360000**	**3.2808610000**	**-0.7198290000**
**H**	**1.2523940000**	**4.6994600000**	**-0.4707670000**
**H**	**2.6692900000**	**3.8698400000**	**-2.3248570000**
**H**	**1.6578750000**	**2.4297800000**	**-2.5026760000**
**H**	**0.0930200000**	**4.1798740000**	**1.6077450000**

**H**	**1.8374690000**	**3.8634240000**	**1.7900680000**
**H**	**-2.9005300000**	**-3.4076860000**	**0.5071250000**
**H**	**-3.8321810000**	**-1.9535920000**	**0.0941880000**
**F**	**-1.5760100000**	**2.0657410000**	**-0.9872450000**
**Cs**	**0.5392770000**	**-0.0252020000**	**-0.7162250000**
**N**	**-2.4763130000**	**1.2473310000**	**1.1049270000**
**C**	**-5.8963370000**	**0.9376700000**	**-0.5006450000**
**C**	**-4.4381680000**	**1.3744960000**	**-0.3932330000**
**C**	**-3.9550790000**	**1.3381590000**	**1.0654600000**
**H**	**-4.2730270000**	**2.2398690000**	**1.5908330000**
**H**	**-2.0720040000**	**1.5506200000**	**1.9841580000**
**H**	**-2.0003800000**	**1.7717960000**	**0.1910360000**
**H**	**-2.2111700000**	**0.2620360000**	**0.9988570000**
**N**	**-6.4570930000**	**0.9966020000**	**-1.8349740000**
**H**	**-5.8215120000**	**0.5436040000**	**-2.4816710000**
**H**	**-6.5569000000**	**1.9576820000**	**-2.1397100000**
**H**	**-5.9619860000**	**-0.0975340000**	**-0.1411860000**
**H**	**-6.5108300000**	**1.5424520000**	**0.1746150000**
**H**	**-4.3727760000**	**0.4722630000**	**1.5884030000**
**O**	**-3.6570070000**	**0.5144260000**	**-1.2003730000**
**H**	**-2.8552030000**	**1.0280750000**	**-1.4213360000**
**H**	**-4.3366140000**	**2.4015300000**	**-0.7666310000**
